# Filling two needs with one deed: a combinatory mucosal vaccine against influenza A virus and respiratory syncytial virus

**DOI:** 10.3389/fimmu.2024.1376395

**Published:** 2024-06-21

**Authors:** Ana Vieira Antão, Friederike Oltmanns, Anna Schmidt, Vera Viherlehto, Pascal Irrgang, Marie-Anne Rameix-Welti, Wibke Bayer, Dennis Lapuente, Matthias Tenbusch

**Affiliations:** ^1^ Institute of Clinical and Molecular Virology, University Hospital Erlangen, Friedrich-Alexander-Universität (FAU) Erlangen-Nürnberg, Erlangen, Germany; ^2^ Université Paris-Saclay – Université de Versailles St. Quentin, UMR 1173 (2I), Institut national de la santé et de la recherche médicale (INSERM), Montigny-le-Bretonneux, France; ^3^ Institute for Virology, University Hospital Essen, University Duisburg-Essen, Essen, Germany; ^4^ FAU Profile Center Immunomedicine (FAU I-MED), Friedrich-Alexander-Universität (FAU) Erlangen-Nürnberg, Erlangen, Germany

**Keywords:** respiratory viruses, mucosal immunity, combinatory vaccine, adjuvant, influenza A virus (IAV), respiratory syncytial virus (RSV)

## Abstract

Influenza A Virus (IAV) and Respiratory Syncytial Virus (RSV) are both responsible for millions of severe respiratory tract infections every year worldwide. Effective vaccines able to prevent transmission and severe disease, are important measures to reduce the burden for the global health system. Despite the strong systemic immune responses induced upon current parental immunizations, this vaccination strategy fails to promote a robust mucosal immune response. Here, we investigated the immunogenicity and efficacy of a mucosal adenoviral vector vaccine to tackle both pathogens simultaneously at their entry site. For this purpose, BALB/c mice were immunized intranasally with adenoviral vectors (Ad) encoding the influenza-derived proteins, hemagglutinin (HA) and nucleoprotein (NP), in combination with an Ad encoding for the RSV fusion (F) protein. The mucosal combinatory vaccine induced neutralizing antibodies as well as local IgA responses against both viruses. Moreover, the vaccine elicited pulmonary CD8^+^ and CD4^+^ tissue resident memory T cells (T_RM_) against the immunodominant epitopes of RSV-F and IAV-NP. Furthermore, the addition of Ad-TGFβ or Ad-CCL17 as mucosal adjuvant enhanced the formation of functional CD8^+^ T_RM_ responses against the conserved IAV-NP. Consequently, the combinatory vaccine not only provided protection against subsequent infections with RSV, but also against heterosubtypic challenges with pH1N1 or H3N2 strains. In conclusion, we present here a potent combinatory vaccine for mucosal applications, which provides protection against two of the most relevant respiratory viruses.

## 1 Introduction

Respiratory viruses, such as IAV and RSV, have a significant impact on global health, mainly among infants and elderly individuals. While the morbidity and mortality rate in adults is similar for both infections (1); infants are significantly affected by RSV, accounting for nearly 33 million infections and up to 120 000 in-hospital deaths under the age of five, every year (2, 3). Vaccines are considered the most cost-effective measure to protect individuals against a wide range of pathogens, including influenza. However, seasonal influenza vaccines only induce strain-specific antibodies against the viral surface proteins, which are rather short-lived and do not provide broad protection against drifted or newly emerging influenza strains, therefore requiring their annual reformulation (4, 5). Considering the risk of new pandemics, efforts are put forward to develop universal influenza vaccines providing broad heterosubtypic immunity, either by T-cells or antibodies directed against conserved epitopes, such as NP or the stalk domain of HA (5, 6).

In case of RSV, vaccine development was hampered for a long time by an early clinical trial with a formalin-inactivated RSV vaccine, in which enhanced RSV disease was observed in immunized children after subsequent natural infection (7, 8). Until recently, passive immunization with monoclonal antibodies targeting the RSV-F protein were the only measure to prevent severe disease (9). However, a better understanding of the mechanism underlying the viral fusion and the discovery of highly immunogenic sites in the prefusion conformation of RSV-F enlighten a new target to improve therapeutic approaches as well as vaccine design (10). Recently, two RSV prefusion F protein-based vaccines were approved for clinical use, still the long-term effectiveness of these vaccine candidates is yet to be unveiled (11, 12).

Finally, the recent pandemic and the intramuscular application of the newly licensed COVID-19 vaccines taught us that systemic immune responses can efficiently protect from severe infection, but breakthrough infections can still occur (13, 14). A reasonable explanation for this phenomenon is the inefficient establishment of mucosal immune responses in the respiratory tract, where these pathogens initiate infection. The presence of pre-existing T_RM_ in the lungs of mice and humans was found to positively correlate with rapid control of viral replication and lower disease burden after influenza and RSV reinfections (15–20). The formation of these cells occurs via the up-regulation of CD69 in activated CD8^+^ and CD4^+^ T-cells and the consequent loss of sphingosine-1-phosphate receptor (S1PR1), which prevents their egression into circulation (21, 22). Moreover, the presence of TGFβ and the suppression of the transcription factors T-bet and Eomes promote the expression of CD103 in CD8^+^ T_RM_, facilitating their adherence to E-cadherin expressed in epithelial cells (23–25). Not only T-cells, but also resident memory B cells (B_RM_) were found in the lungs of influenza-infected mice, which allowed a faster antibody response upon a secondary challenge (26, 27). In contrast to T_RM_, these cells do not express CD103 and are usually characterized by the expression of CD69 and the chemokine receptors CCR6 and CXCR3 (27, 28). Their formation is thought to occur in inducible bronchoalveolar lymphoid tissues (iBALT), where they are long-lived together with CD4^+^ T_RM_ and CD11c^+^ dendritic cells (DCs) (27, 29, 30). Thereby, mucosal vaccines able to elicit long-lasting and protective T_RM_ and B_RM_ responses against respiratory viruses are considered promising candidates. Among the different vaccine platforms available, viral vectors and specifically adenoviral vectors are considered excellent candidates to deliver vaccine antigens due to their ability to induce robust T-cell and antibody responses (31). In addition to finding a suitable vaccine platform, the usage of innate endogenous molecules as adjuvants have also been considered relevant to improve the immunogenicity and efficacy of mucosal vaccines (32). Recently, we have demonstrated that the intranasal administration of an Ad5-based vaccine targeting the HA and NP proteins of IAV together with an Ad5-expressing Interleukin-1β (Ad-IL1β) as a mucosal adjuvant, elicited a superior amount of CD8^+^ T_RM_ in the lungs and enhanced vaccine efficacy against heterologous influenza strains in comparison to the non-adjuvanted treatment (33). Similar observations were made using Ad-IL1β in combination with an Ad5 expressing the fusion protein of RSV (34). Although the mucosal administration of IL1β revealed to enhance vaccine immunogenicity and efficacy, its pleiotropic effect can elicit undesired inflammation. Here, we evaluated CCL17 and TGFβ, two other molecules which have been described to have a direct impact on the formation of T_RM_ in the lung. While CCL17 is involved in the recruitment of T-cells via the interaction with the chemokine receptor CCR4, TGFβ promotes the upregulation of CD103 and thereby tissue retention of CD8^+^ T-cells (23, 35, 36).

In this study, we evaluated the immunogenicity and efficacy of a mucosal combinatory vaccine composed of Ad5-vectors encoding the IAV-HA, IAV-NP and RSV-F proteins (Ad-HA/NP/F) in combination with the adjuvants Ad-TGFβ and Ad-CCL17. We could demonstrate that this vaccine approach efficiently induced local cellular and humoral responses against both viruses providing protection from subsequent infection with RSV or two distinct IAV strains. Furthermore, the co-delivery of Ad-TGFβ or Ad-CCL17 as adjuvants showed potential to further enhance the formation of vaccine-specific CD8^+^ and CD4^+^ T_RM._

## 2 Material and methods

### 2.1 Adenoviral vectors

The adenoviral vectors vaccines and adjuvants are replication-deficient (ΔE1ΔE3) and based on the human serotype 5 (Ad5). Ad-HA and Ad-NP vaccines were generated from a codon-optimized gene sequence derived from A/H1N1/Puerto Rico/8/1934 (PR8) as described previously (33). The vaccine Ad-F was produced from a codon-optimized gene sequence from the RSV-A2 F protein as described before (37). High-titer viral stocks of the vaccines and the control vector lacking the transgene expression (Ad-Empty) were obtained in collaboration with Sirion Biotech (Martinsried, Germany). The established AdEasy system was used to obtain the mucosal adjuvants, Ad-TGFβ and Ad-CCL17. The respective ORFs were cloned into the vector pShuttle-CMV, which was then used for homologous recombination with the adenoviral vector pAdEasy-1 (38). The recombinant viruses were propagated in HEK 293 cells and viral particles (vp) were purified and concentrated with the Vivapure Adenopack20 (Sartorius). The concentration of total Ad was measured by optical density at 260 nm (OD_260_) and the infectious particles by Reed-and-Muench TCID_50_ (39). Ratios of total to infectious particles were typically bellow 200:1.

### 2.2 Mice and immunizations

Six to eight weeks old female BALB/cJRj mice were purchased from Janvier (Le Genest-Saint-Isle, France) and kept in individually ventilated cages in accordance with German law and institutional guidelines under pathogen-free (SPF) condition, with constant temperature (20–24°C) and humidity (45–65%) on a 12 h/12 h-light/dark cycle. The research staff was trained in animal care and handling in accordance to the FELASA and GV-SOLAS guidelines. The study was approved by the Government of Lower Franconia, which nominated an external ethics committee that authorized the experiments. Studies were performed under the project license AZ. 55.2.2–2532-2–1085. Mice were intranasally immunized with a dose of 2x10^8^ vp (calculated from optical density measurement) of each antigen-encoding vector (Ad-HA, Ad-NP and Ad-F) in combination with 1x10^9^ vp encoding the adjuvant (Ad-TGFβ, Ad-CCL17 or Ad-empty). The vaccine was slowly pipetted in a total volume of 50 µl into one nostril under general anesthesia (100 mg/kg ketamine and 15 mg/kg xylazine). In all experiments, unvaccinated animals served as naïve negative control to define background levels. Blood samples were collected from the retro-orbital sinus under light anesthesia with inhaled isoflurane. Animals were euthanized with isoflurane and bronchoalveolar lavage fluids (BAL) were collected by washing the lungs with 2x1 ml PBS 1x via the cannulated tracheae. In addition, lungs and spleens were removed for further analysis.

### 2.3 Antigen-specific antibody ELISA

96-well ELISA plates (Lumitrac, high binding, Greiner Bio-One) were coated with heat-inactivated (30 min, 56°C) 5x10^5^ PFU/well PR8 or RSV-A2 diluted in carbonate buffer overnight at 4°C. Afterwards, free binding sites were blocked with 5% skimmed milk in PBS-T_0.05_ (PBS containing 0.05% Tween-20, Sigma-Aldrich). After a washing step with PBS-T_0.05_, diluted sera or BAL were added and incubated for one hour at room temperature. Subsequently, plates were washed and the detection antibodies, HRP-coupled polyclonal anti-mouse IgG (1:3000, PA1–84631, Invitrogen) or anti-mouse IgA (1:5000, A90–103P, Bethyl Laboratories), were added for one hour. After washing with PBS-T_0.05_ and the addition of an ECL substrate, the signals were acquired on a microplate luminometer (VICTOR X5, Perkin Elmer) with the PerkinElmer 2030 Manager software.

### 2.4 Influenza microneutralization assay

Neutralizing antibody titers against influenza virus were determined by a microneutralization assay as previously described (40). A two-fold serial dilution of complement-inactivated (56°C, 30 min) serum samples were incubated with 2000 PFU of PR8. The samples and the virus were diluted in Dulbecco’s modified Eagle’s medium containing 0.6% ml BSA, 100 units/ml penicillin/streptomycin, and 1.2 µl/ml Trypsin. Afterwards, the serum-virus mix was added on top of confluent MDCK II cells in a 96-well plate. Four days later, plaques were identified by crystal violet staining. The neutralization titer corresponded to the highest dilution in which complete inhibition of infectivity was observed.

### 2.5 RSV microneutralization assay

RSV-neutralizing antibody titers were determined using a luciferase-based microneutralization assay. Two-fold serial dilution of complement-inactivated serum samples were incubated with 2.55x10^4^ TCID_50_ of a recombinant RSV-A2 virus expressing firefly luciferase (41). The serum-virus mix was then added onto confluent Hep2 cells, previously seeded in a 96-well plate with serum-free DMEM (2 mM L-Glutamine, 100 units/ml penicillin/streptomycin). Four hours after incubation at 37°C, 2% DMEM was added to the cells (2% FCS, 2 mM L-Glutamine, 100 units/ml penicillin/streptomycin). After 24 h at 37°C, cell lysis was performed with Glo Lysis Buffer 1x (Promega) for 15 min at 37°C. Luciferase luminescence was detected after 3 min incubation with Bright-Glo™ Luciferase Assay System (Promega). The plates were acquired on a microplate luminometer (VICTOR X5, PerkinElmer) using PerkinElmer 2030 Manager Software. The 50% plaque reduction neutralization titers (PRNT50) were defined as the highest dilution that inhibited more than 50% of plaques observed in eight infected control wells without serum treatment.

### 2.6 FACS-based antibody analysis

HEK 293A cell lines expressing viral antigens under the control of a doxycycline-inducible promoter were generated after stable transduction with lentiviral particles encoding either the F protein of RSV-A2 (34), the HA or the NP protein of PR8. Initially, target cells were treated for 24–48 h with doxycycline (100–400 ng/ml) to induce antigen expression. Afterwards, 1x10^5^ cells were plated in a 96-well plate and HA- and F-producing HEK 293A cells were incubated with sera and BAL samples diluted in FACS buffer (PBS with 0.5% BSA and 1 mM sodium-azide) for 30 min at 4°C. Antibodies specific for intracellular NP were analyzed after incubation of permeabilized cells (0.5% saponin in FACS buffer) with samples diluted in permeabilization buffer. Bound HA-, NP- and F-specific antibodies were detected with either a polyclonal anti-mouse IgG-FITC (1:300, 406001, BioLegend) or a polyclonal anti-mouse IgA-FITC (1:300, A90–103F, Bethyl Laboratories) diluted in either FACS or permeabilization buffer. After incubation with the secondary antibodies for 20 min at 4°C, HA- and F-producing cells were fixated (2% PFA in PBS, 1004965000, Sigma-Aldrich). Samples were acquired on an Attune NxT (ThermoFisher) and analyzed with FlowJo™ software (Treestar Inc.).

### 2.7 Intracellular cytokine staining

Fifty-two days after immunization, mice were injected intravenously with 2 µg of anti-CD45-BV510 (clone 30-F11, BioLegend) to distinguish circulating from resident T-cells and sacrificed 3 min later with isoflurane. Lungs were collected and lymphocytes obtained after incubation of cut lung tissue pieces with 500 units Collagenase D (Sigma-Aldrich) and 160 units DNase I (Applichem) in 2 ml R10 medium (RPMI 1640 supplemented with 10% FCS, 2 mM L-Glutamine, 10 mM HEPES, 50 μM β-Mercaptoethanol and 1% penicillin/streptomycin) for 45 min at 37°C. Once digested, lung pieces were homogenized through a 70 µm cell strainer and incubated with an ammonium-chloride-potassium buffer (Gibco) to lyse erythrocytes. In the end, one eighth of the total lung cell suspension was plated in a 96-well round-bottom plate and incubated for 6 h with 100 µl of R10 plus monensin (2 µM), anti-CD28 (1 µg/ml, eBioscience), anti-CD107a-FITC (1:100, clone 1D4B, BD Biosciences) and 5 µg/ml of the respective MHC-I peptides (Genescript); NP_147_-_155_ TYQRTRALV or a F-pool (F_80-94_ KQELDKYKNAVTEKQ; F_84-98_ DKYKNAVTELQLLMQ; F_243-257_ DKYKNAVTELQLLMQ; F_247-261_ VSTYMLTNSELLSLI) to analyze T-cell cytokine expression. After restimulation, cells were stained with anti-CD8a-Pacific blue (1:300, clone 53–6.7, BioLegend), anti-CD4-PerCP eFluor710 (1:2000, clone RM4–5, eBioscience) and Fixable Viability Dye eFluor^®^ 780 (1:100, eBioscience). Subsequently, cells were washed with FACS buffer, fixed in 2% PFA and permeabilized with 0.5% saponin in FACS buffer. Cells were then stained intracellularly with anti-IL-2-APC (1:300, clone JES6-5H4, BioLegend), anti-TNFα-PECy7 (1:300, clone MPG-XT22, BioLegend) and anti-IFNy-PE (1:300, clone XMG1.2, BioLegend) diluted in permeabilization buffer. Data was acquired on Attune NxT (ThermoFisher) and analyzed with FlowJo™ software (Treestar Inc.).

### 2.8 Phenotypic T-cell analysis

Lymphocytes were obtained as described in the previous section and stained with either APC-labelled H-2K^D^ NP_147-155_ (1:40, TYQRTRALV, ProImmune) or H-2K^D^ F_85_-_93_ (1:40, KYKNAVTEL, ProImmune) pentamers, followed by a second step staining with anti-CD69-BV421 (1:100, clone H1.2F3, BioLegend), anti-CD4-BV605 (1:1000, clone RM4–5, BioLegend), anti-CD19-BV650 (1:300, clone 6D5, BioLegend), anti-CD8-BV711 (1:500, clone 53–6.7, BioLegend), anti-CD49a-BV785 (1:300, clone Ha31/8, BD Biosciences), anti-CD45-FITC (1:300, clone 104, BioLegend), anti-CD11a-PerCP eFluor 710 (1:300, clone M17/4, Invitrogen), anti-CD103-PE (1:100, clone 2E7, Invitrogen), anti-CD127-PE/Dazzle (1:300, clone A7R34, BioLegend), anti-CD44-PECy5 (1:2000, clone IM7, BioLegend), anti-KLRG1-PECy7 (1:300, clone 2F1, Invitrogen) and Fixable Viability Dye eFluor^®^ 780 (1:100, eBioscience). Data was acquired on Northern Lights (Cytek) and analyzed with FlowJo™ software (Treestar Inc.).

### 2.9 Influenza infections

Fifty- six days after vaccination, mice under general anesthesia (100 mg/kg ketamine and 15 mg/kg xylazine) were inoculated intranasally with 2000 PFU (2–4 x LD50) of mouse-adapted A/pH1N1/Hamburg/4/2009 (pH1N1) (42) or mouse-adapted A/H3N2/Hong Kong/1/1968 (H3N2, kindly provided by Prof. Georg Kochs, University Hospital Freiburg, Germany) in a final volume of 50 µl PBS. Weight loss and clinical score were monitored daily throughout infection and mice were euthanized when reaching the humane endpoint criteria. Lungs were collected and then homogenized in M-tubes with a GentleMACS dissociator (Miltenyi Biotec). Viral RNA was isolated from cell-free lung and BAL supernatants using the NucleoSpin^®^ RNA Virus kit (Machery-Nagel) according to the manufacturer’s instructions. Viral replication was quantified by SYBR-Green quantitative reverse transcriptase real-time PCR (qRT-PCR) using the GoTaq^®^ RT-qPCR 1-Step Kit (Promega) or TaqMan™ qRT-PCR using the AgPath-ID One-Step RT-PCR Kit (Thermofisher) with a 5’ FAM/3’ BHQ-1 probe (tcaggccccctcaaagccga). The universal primers used amplify the influenza matrixprotein 2 gene (forward: agatgagtcttctaaccgaggtcg, reverse1: tgcaaaaacatcttcaagtctctg, reverse2: tgcaaagacatcttccagtctctg). The data were log10-transformed and the lower limit of quantification was at 666 copies/ml. Tissue damage was determined by detecting total protein in cell-free BAL using bicinchoninic acid assay (Pierce) according to the manufacturer’s instructions.

### 2.10 RSV infection

RSV challenge was conducted by the intranasal inoculation of mice under general anesthesia (100 mg/kg ketamine and 15 mg/kg xylazine) with 5x10^5^ PFU of RSV-A2 (ATCC VR-1540) in a final volume of 50 µl PBS. Body weight and clinical score were monitored daily throughout infection. Mice were euthanized five days post infection. Lungs were collected and then homogenized in M-tubes with a GentleMACS dissociator (Miltenyi Biotec). Viral RNA was isolated from cell-free lung supernatants using the NucleoSpin^®^ RNA Virus kit (Machery-Nagel) according to the manufacturer’s instructions. Viral replication was quantified by SYBR-Green qRT-PCR using the GoTaq^®^ RT-qPCR 1-Step Kit (Promega) with primers for a sequence in the nucleoprotein gene (forward: agatcaacttctgtcatccagcaa, reverse: gcacatcataattaggagtatcaat). The data were log10-transformed and the lower limit of quantification was at 666 copies/ml. Tissue damage was determined by detecting total protein in cell-free BAL using bicinchoninic acid assay (Pierce) according to the manufacturer’s instructions.

### 2.11 Statistical analysis

All data sets were tested for normal distribution by using Shapiro-Wilk Test. Passing the normality test, one-way ANOVA or Two-way ANOVA with Tukey’s posttest were performed, otherwise, a non-parametric Kuskal Wallis Test with Dunn´s multiple comparisons was used. A p value of <0.05 was considered to be statistically significant. All analyses were done with Prism 9.0 (GraphPad Software, Inc.).

## 3 Results

### 3.1 The combinatory mucosal vaccine induces both systemic and mucosal antibodies

**Figure 1:**
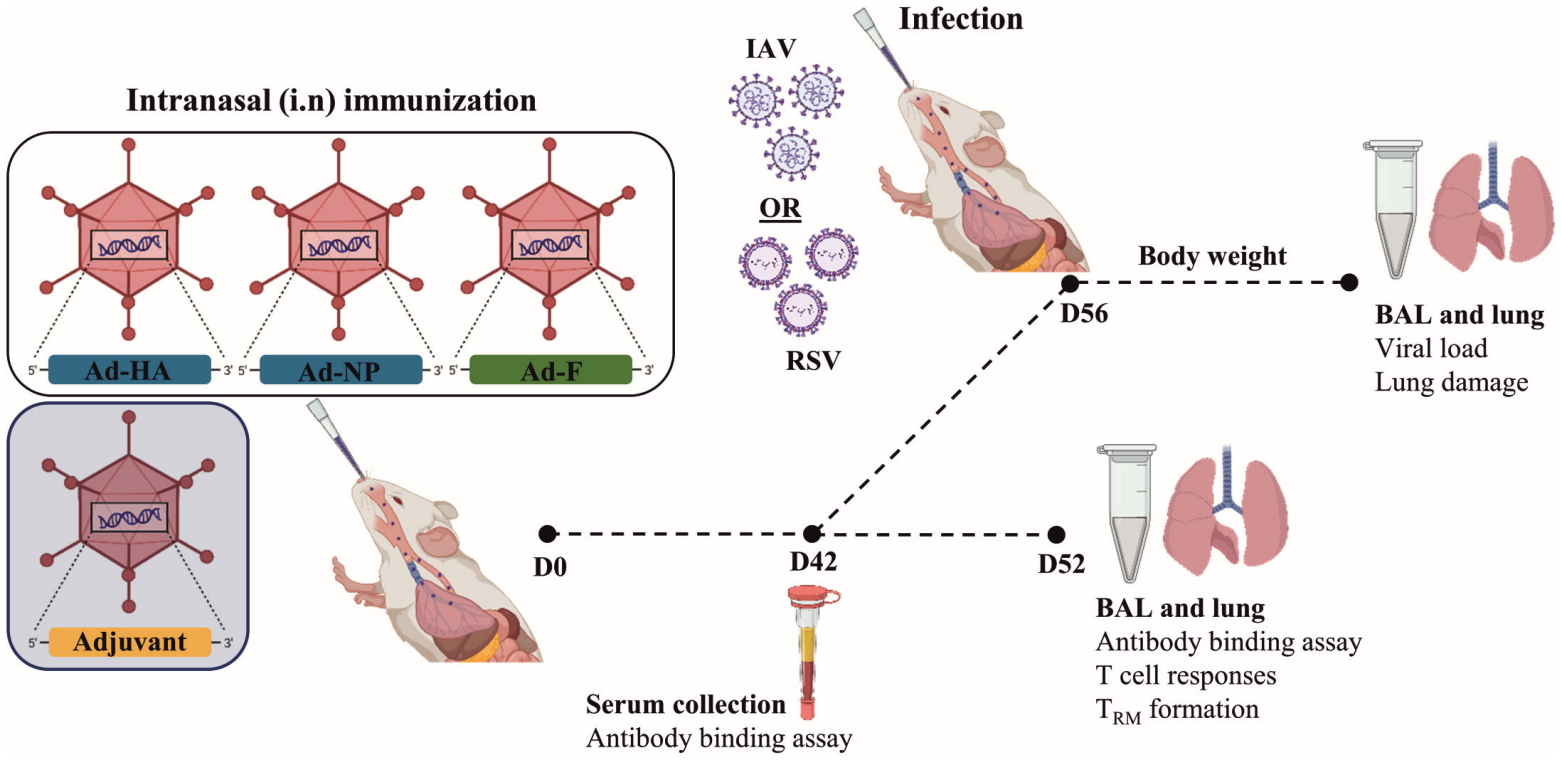
Experimental scheme to study the immunogenicity of the different adjuvants. At day 0, mice were immunized intranasally with 2x10^8^ vp of Ad-HA/NP/F, together with 1x10^9^ vp of the adjuvant. Forty-two days after immunization, blood was collected and humoral responses were analyzed in the serum. Four to six animals per group were euthanized at day 52 to investigate T-cell phenotype and responses in the lungs. In addition, bronchoalveolar lavages (BAL) were used to determine mucosal antibody responses. Another six to eight animals per group were infected with 2000 PFU of H3N2 or 5x10^6^ PFU of RSV-A2 to determine vaccine efficacy. Weight loss was monitored every day. The end of the experiment was determined when a 20% weight loss was observed. BAL and lungs were collected to determine viral load and lung damage. Created with BioRender.com.

Vaccine immunogenicity was determined after intranasal immunization of BALB/c mice with an Ad5-based vaccine encoding for the HA and NP proteins of PR8 and RSV-F protein (Ad-HA/NP/F) in combination with either Ad-TGFβ or Ad-CCL17 as mucosal adjuvants (**Figure 1**). As a control group, we used mice vaccinated with Ad-HA/NP/F plus an empty Ad5-vector construct to standardize the amount of particles each mouse received (Ad-Empty). A second control group consisted of naïve, non-vaccinated animals to validate background levels in the immunological assays. None of the mice showed any signs of adverse effects and histological analyses of the lung did not reveal signs of tissue damage or extensive inflammation two weeks after immunization (not shown).

**Figure 2:**
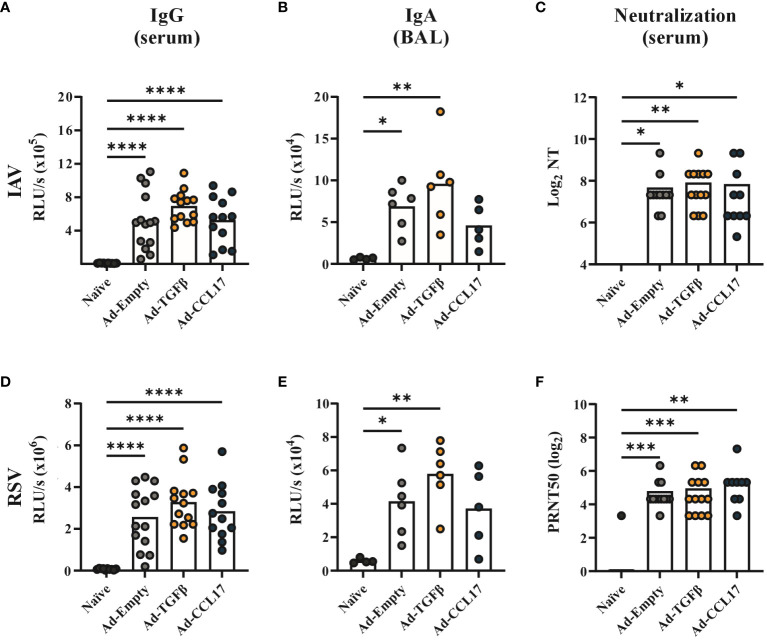
Systemic and humoral antibody responses after vaccination. BALB/c mice were immunized with 2x10^8^ vp of Ad-HA, Ad-NP and Ad-F plus 1x10^9^ vp of either Ad-Empty, Ad-TGFβ or Ad-CCL17. Serum and BAL samples were collected forty-two and fifty-two days after immunization, respectively. IAV- (upper panel) or RSV- (lower panel) specific antibody responses were analyzed in the serum and BAL. For ELISA measurement, plates coated with either inactivated PR8 or RSV-A2 were used to quantify virus-specific IgG in serum (**A, D**, dilution 1:500) or IgA in BAL (**B, E**, dilution 1:50). Neutralizing antibodies against PR8 were detected in serum samples by a microneutralization assay **(C)**. 50% plaque reduction neutralization titers (PRNT50) were analyzed in serum samples by RSV neutralization assay **(F)**. Results represent 10 to 14 mice per group for serum samples and four to six for BAL samples. Each dot represents an individual animal and bars the corresponding mean. Statistical significances were analyzed by one-way ANOVA followed by Tukey’s post test **(A, B, D, E)** or by Kruskal Wallis Test with Dunn´s multiple comparisons test **(C, F)**. *p<0.05; **p<0.01; ***p<0.001; ****p<0.0001.

Using whole virus particles as coating agent, we detected IAV- and RSV-specific IgG antibodies in serum (**Figures 2A, D**) 42 days and IgA antibodies in BAL (**Figures 2B, E**) 52 days after a single shot of the combinatory vaccine. Furthermore, the neutralizing capacity of sera was confirmed in IAV-and RSV-specific neutralization assays (**Figures 2C, F**). In an independent flow cytometry based assay, IgG and IgA antibodies against all three antigens could also be detected, however none of the adjuvants significantly impacted vaccine-induced antibody responses (**Supplementary Figure S1**). Nevertheless, IgA responses to HA and RSV-F were slightly higher after co-administration of Ad-TGFβ (**Supplementary Figure S1**).

### 3.2 The combinatory mucosal vaccine induces CD4 T_RM_ and B_RM_

Next, we performed a phenotypic analysis of the lung-derived lymphocytes and investigated the effect of the immunization strategy on the development of mucosal CD4^+^ T- and B-cells. To distinguish circulating from resident lymphocytes, a fluorescent anti-CD45 antibody was administered intravenously before sacrifice. Since resident T- and B-cells do not circulate, these cells are protected from the intravenous staining and therefore are considered CD45 iv^-^ (gating strategy shown in **Supplementary Figure S2**).

**Figure 3:**
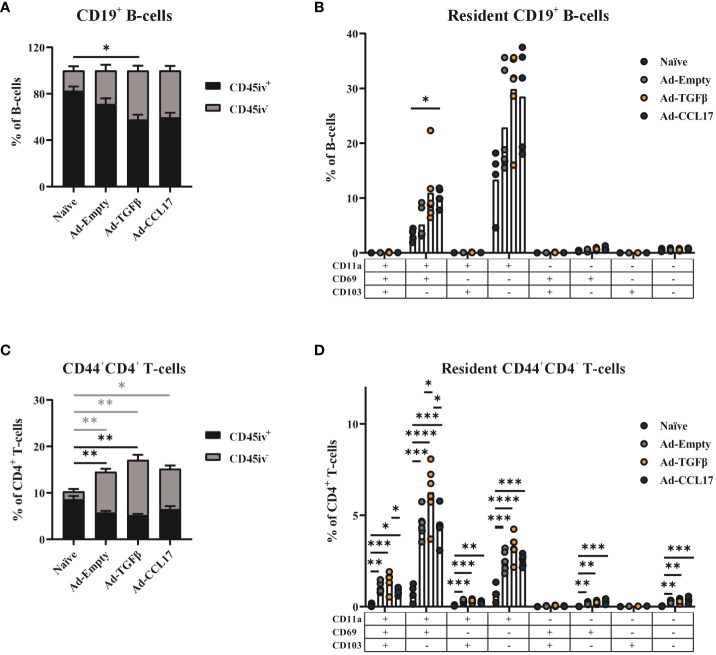
Phenotypic analysis of resident memory B-cells and CD4^+^ T-cells. Mice were immunized as described before and 56 days later, lymphocytes were isolated from lung tissue. Circulating (CD45 iv^+^) and resident (CD45 iv^-^) lymphocytes were distinguished by the intravenous staining with anti-CD45, before sacrificing mice. The frequency of resident and circulating cells was determined in total CD19^+^ B-cells **(A)** or CD44^+^CD4^+^ T-cells **(C)**. The different phenotypes of the CD45 iv^-^ compartment were characterized by CD11a, CD69 and CD103 staining. The frequency of the different combinations was determined in total B-cells **(B)** or CD4^+^ T-cells **(D)**. Each dot represents an individual animal and bars the corresponding mean. Statistical significances were analyzed by Kruskal Wallis Test with Dunn´s multiple comparisons test **(A–C)** or by one-way ANOVA followed by Tukey’s post test **(C–E)** or. *p<0.05; **p<0.01; ***p<0.001; ****p<0.0001.

In the B-cell compartment of the vaccinated animals, higher proportions of CD45 iv^-^ cells (30–50%) were detected compared to the naïve controls (less than 20% of all B-cells; **Figure 3A**). Since we can not specify the antigen-reactivity of those B-cells, the high proportion of iv^-^ cells in the naïve group might indicate some limitations of the intravascular staining for the analyses of B_RM_. Nevertheless, to characterize the B_RM_ compartment in more detail, we performed an analysis using the markers CD11a, CD69 and CD103 within the CD19^+^ iv^-^ population (gating strategy shown in **Supplementary Figure S2**). In infection settings, B_RM_ have been described to not express CD103 (27). Here we investigated whether similar phenotypes were found in the vaccine context. Indeed, CD103 expression was also not found in B_RM_ induced after mucosal immunization with Ad-HA/NP/F. Most of the CD45 iv^-^ B-cells found in the lungs of mice express the integrin CD11a, but lack expression of CD69 and CD103. This B-cell population was increased upon vaccination, while the mucosal adjuvants seem to further promote its formation (**Figure 3B**). Furthermore, a second CD11a^+^ B_RM_ population expressing additionally the activation marker CD69 was found at the highest frequencies in the adjuvanted mice (**Figure 3B**), but the individual variability within the groups was too high to reach statistical significant differences.

The presence of vaccine–induced, antigen-experienced CD4^+^ T_RM_ was also assessed in the lungs of mice via the surface staining with anti-CD44 in combination with the intravenous staining (gating strategy shown in **Supplementary Figure S2**). Although the overall proportion of CD44^+^ cells among lung-derived CD4^+^ T-cells was already increased in all vaccine groups, statistical significance was reached when considering specifically the CD45 iv^-^ compartment (**Figure 3C**).

Similarly to the B_RM_ analyses, the presence of CD11a, CD69 and CD103 were used for a detailed characterization of the CD4^+^ T_RM_ population (gating strategy shown in **Supplementary Figure S2**).

Here, a heterogeneous T_RM_ profile was induced by vaccination and a small percentage of cells expressing CD103 was also detected (**Figure 3D**). However, CD11a^+^CD69^+^CD103^-^ was the most predominant phenotype found among CD4^+^ T_RM_, followed by the single expression of CD11a. The percentage of these T_RM_ phenotypes was significantly increased upon vaccination, and the mucosal adjuvant Ad-TGFβ seemed to have a superior effect on their formation compared to Ad-CCL17-adjuvanted or the non-adjuvanted vaccine. Since TGFβ is not only important for T_RM_ formation, but also involved in the development of regulatory CD4^+^ T cells (T_reg_), we also quantified Foxp3^+^CD25^+^ CD4^+^ T-cells in the lungs of immunized mice (gating strategy shown in **Supplementary Figure S3B**). Even though, compared to the naïve animals, frequencies were increased and reached statistical significance for the two adjuvanted groups, there was no significant difference in the frequency of T_reg_ among the vaccine groups (**Supplementary Figure S3C**). Therefore, the mucosal delivery of TGFβ and CCL17 did not seem to further increase the frequency of vaccine induced T_reg_ in the lungs of mice.

### 3.3 The combinatory mucosal vaccine induces functional NP- and F-specific CD8^+^ T_RM_

**Figure 4:**
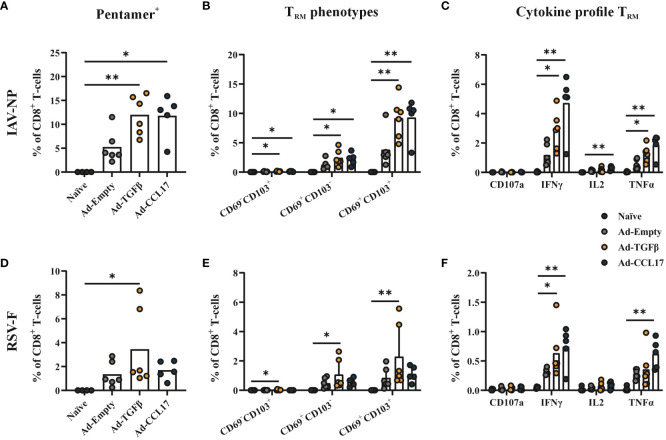
Antiviral CD8^+^ T_RM_. Mice were immunized as described before and 56 days later, lymphocytes were isolated from lung tissue. Resident memory CD8^+^ T-cells were identified by the absence of the intravenous labeling with anti-CD45 (CD45 iv^-^) and by MHC-I pentamers loaded with NP- (upper panel) or F- (lower panel) peptides. The frequencies of pentamer-specific CD8^+^ T_RM_ **(A, D)** and the different T_RM_ phenotypes based on the expression of CD69 and CD103 **(B, E)** were determined in total CD8^+^ T-cells. Functional antiviral CD8^+^ T-cells were analyzed via intracellular cytokine staining after *in vitro* restimulation with NP- or F-specific peptides **(C, F)**. The proportion of CD107a^+^, IFNγ^+^, IL-2^+^ and TNFα^+^ CD8^+^ T-cells among all CD8^+^ T-cells were shown. Each dot represents an individual animal and bars the corresponding mean. Statistical significances were analyzed by Kruskal Wallis Test with Dunn´s multiple comparisons test. *p<0.05; **p<0.01.

Next, we investigated the effect of the mucosal vaccination on the development of CD8^+^ T_RM_, as these cells are considered the first line of defense against respiratory tract infections. Circulating and resident cells were distinguished by an intravenous labeling with anti-CD45 and then further characterized with APC-labeled MHC-I pentamers loaded with either the NP_147-155_ or F_85-93_ peptides (gating strategy shown in **Supplementary Figure S2**). The intranasal immunization with Ad-HA/NP/F significantly increased the presence of total CD8^+^ T-cells in the lungs of mice, which was further enhanced in combination with either Ad-TGFβ or Ad-CCL17 (**Supplementary Figure S4A**). Within this T-cell compartment, we could detect both NP- and F-specific CD8^+^ T-cells after mucosal immunization (**Figures 4A, D**), which belonged almost exclusively to the resident compartment (**Supplementary Figures S4B, C**). The administration of either Ad-TGFβ or Ad-CCL17, as mucosal adjuvants, significantly increased the frequencies of NP-specific CD8^+^ T_RM_ in comparison to the naïve group (**Figure 4A**). However, only the adjuvant Ad-TGFβ had a slight impact on the formation of F-specific CD8^+^ T_RM_ (**Figure 4D**). Here, a detailed analysis of the T_RM_ phenotype was also performed via the surface staining with CD69 and CD103 (gating strategy shown in **Supplementary Figure S2**). Although most of the NP- and F-specific CD8^+^ T_RM_ expressed CD69 and CD103, a fraction of these cells was also found to be CD69^+^CD103^-^ (**Figures 4B, E**). The frequency of CD69^+^CD103^+^ NP-specific CD8^+^ T_RM_ was significantly increased after the mucosal immunization with the adjuvanted vaccines, but only Ad-TGFβ increased vaccine-induced CD69^+^CD103^+^ F-specific CD8^+^ T_RM_.

Beside the phenotypic analysis, resident CD8^+^ T-cell responses were also functionally analyzed by intracellular cytokine staining. Lymphocytes isolated from the lungs of mice were restimulated with MHC-I restricted NP and F peptides, followed by the detection of IFNγ, TNFα and IL-2 as well as the granulation marker CD107a within this CD8^+^ T-cell population (**Supplementary Figure S5**). In all immunized groups, IFNγ- and TNFα–producing CD8^+^ T_RM_ were observed upon *in vitro* restimulation of lung lymphocytes, with NP- and F-specific peptides, and this was further increased when using the mucosal adjuvants (**Figures 4C, F**).

### 3.4 The combinatory mucosal vaccine protects against RSV infection

**Figure 5:**
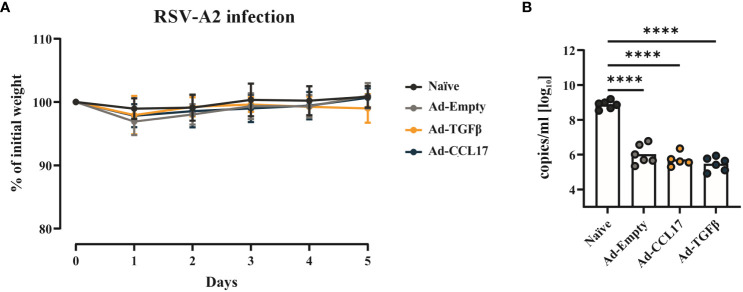
Vaccine efficacy against RSV. BALB/c mice were immunized as described before and infected with 5x10^6^ PFU of RSV-A. Weight loss after infection was monitored daily and all the animals were killed when the naïve group reached the endpoint criteria **(A)**. Viral RNA **(B)** was determined in the lungs of mice via qRT-PCR. Each dot represents an individual animal and bars the corresponding mean. Statistical significances in weight loss were analyzed by two-way ANOVA followed by Tukey’s post test (n=5–6). Differences in viral loads were tested for statistical significances by one-way ANOVA followed by Tukey’s post test (n=5–6). ****p<0.0001.

After investigating vaccine immunogenicity, we also determined its protective effect against RSV. For this purpose, fifty-six days after vaccination, mice were infected with 5x10^6^ PFU of RSV-A2 (**Figure 1**). Body weight was measured throughout infection, however no substantial weight loss was observed (**Figure 5A**). Five days after challenge, animals were euthanized and viral load was measured in BAL samples. Immunized mice showed significantly lower copy numbers of viral RNA compared to the naïve group (**Figure 5B**). However, no statistical differences were observed between non-adjuvanted and adjuvanted animals.

### 3.5 The combinatory mucosal vaccine protects against heterosubtypic IAV challenges

**Figure 6:**
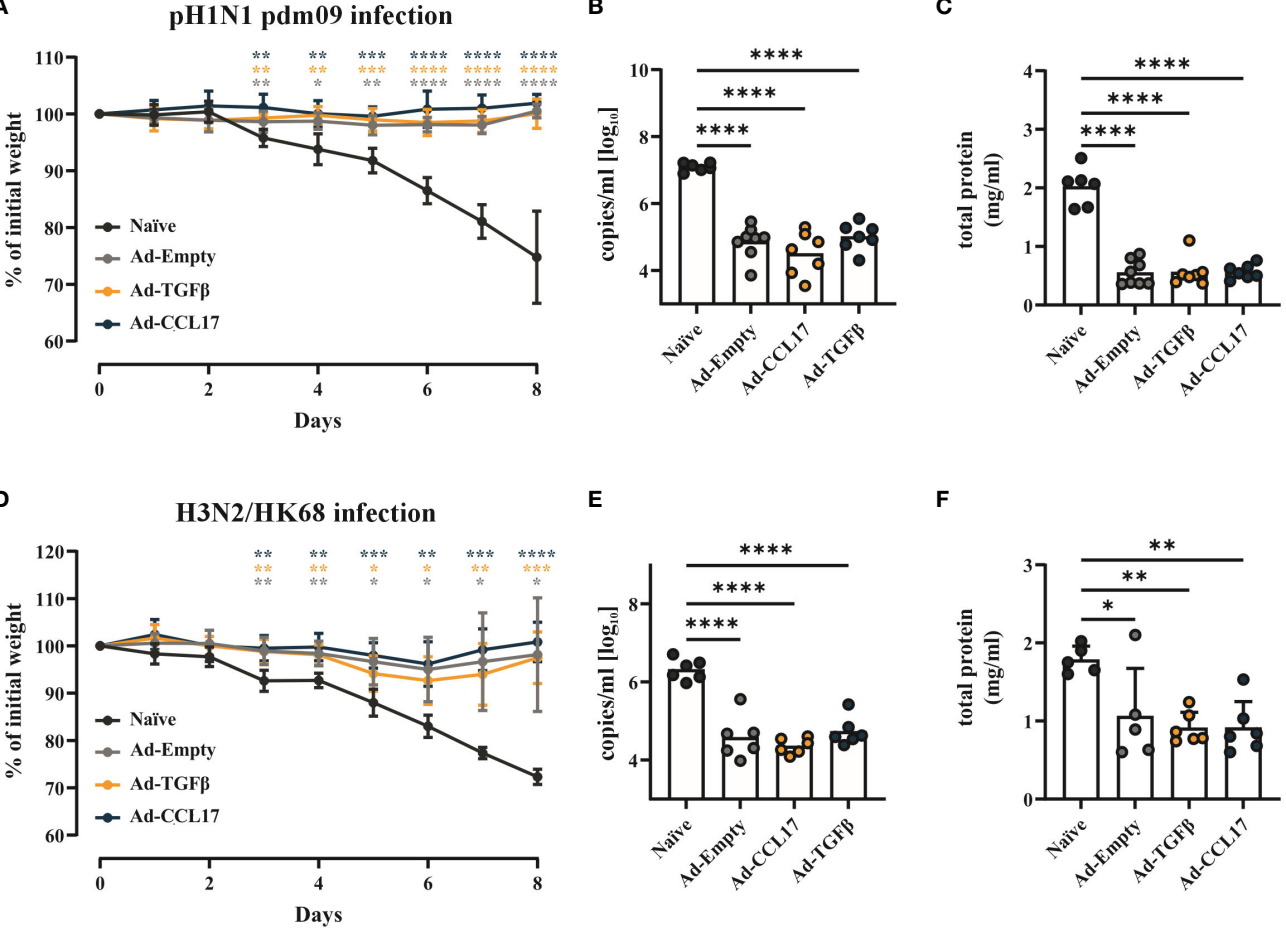
Vaccine efficacy against heterosubtypic IAV strains. BALB/c mice were immunized as described before and infected with either 2000 PFU of pH1N1 or H3N2. Weight loss after infection was monitored daily and all the animals were euthanized when the naïve group reached the endpoint criteria **(A, D)**. Viral RNA copy numbers **(B, E)** were determined in the lungs and the total amount of protein **(C, F)** was analyzed in the BAL of mice. Each dot represents an individual animal and bars the corresponding mean. Statistical significances in weight loss were analyzed by two-way ANOVA followed by Tukey’s post test and p values were determined vs. the naïve group (n=5–8). Viral load and total amount of protein were tested for statistical significances by one-way ANOVA followed by Tukey’s post test (n=5–8). *p<0.05; **p<0.01; ***p<0.001; ****p<0.0001.

Next, we assessed vaccine efficacy against two different heterologous IAV strains. Fifty-six days after vaccination, mice were infected with 2000 PFU of either pH1N1 or H3N2 (**Figure 1**). Body weight was measured during infection and, at day 8 viral load and lung damage were determined. Independently of the adjuvants, the mucosal immunization with Ad-HA/NP/F protected mice against weight loss upon infection with the pH1N1 (**Figure 6A**). In contrast, unimmunized mice had already lost 4.2% ± 1.5% of their initial weight three days post infection (p.i.) and continuously decreased until the experimental endpoint was reached on day 8 (25.2% ± 8.1%; **Figure 6A**). Accordingly, all immunized mice had a significant reduction in viral RNA levels (**Figure 6B**). Furthermore, the amount of protein found in the BAL was highly reduced in the vaccinated animals indicating reduced damage of lung tissue (**Figure 6C**).

The challenge with the more distant H3N2 strain led to a symptomatic infection also in the immunized groups, since all animals showed slight weight loss three days p.i. (**Figure 6D**). Nevertheless, all vaccinated animals regained weight starting at day 7 and recovered from disease, whereas naïve mice reached the humane endpoint at day 8 p.i. (**Figure 6D**). In line with this attenuated disease phenotype, significant reductions in viral loads and protein levels were found in the BAL of immunized animals (**Figures 6E, F**).

## 4 Discussion

Vaccines are the most cost-effective method available to prevent the global disease burden caused by viral infections (5, 43). However, only recently two RSV vaccines were licensed for clinical use and seasonal influenza vaccines are known to provide only short-lived immunity against a very narrow range of IAV strains. Although they are essential to reduce viral spread and control disease severity, the strain-specific antibodies elicited after influenza vaccination are not effective against new emerging influenza strains (4, 44). Additionally, most of these vaccines do not promote local immune responses against these respiratory viruses, such as the formation of T_RM_ which have been found in influenza and RSV-infected individuals to reduce disease severity (15, 19).

Live-attenuated vaccines are the only influenza vaccine licensed to be administered intranasally and able to promote a strong mucosal IgA and broader T-cell responses (45). Moreover, in the mouse model, this vaccine was observed to elicit the formation of protective CD8^+^ and CD4^+^ T_RM_ in the lungs (46). Because the intranasal application results in self-limiting infection of the upper respiratory tract, live-attenuated influenza vaccines (LAIV) can induce a robust mucosal immunity against viral infection. However, low efficacy against the 2009 H1N1 pandemic strain has been reported in young individuals and the possibility of recombination with circulating strains is a major safety concern when considering this vaccine platform (44, 47, 48). For this reason, new immunization strategies need to be explored to overcome the drawbacks from current licensed vaccines. In the present study, we investigated a mucosal combinatory vaccine against IAV and RSV able to induce neutralizing antibody responses against the respective pathogens as well as resident T- and B-cell populations. Moreover, the induction of mucosal immune responses after vaccination led to the protection of mice against IAV and RSV infections.

Combinatory vaccines are routinely administered in pediatric immunization schedules. Besides causing less trauma to the infant due to the reduced number of immunizations, better vaccine coverage has also been observed (49, 50). Since, the development of the first combined vaccine against diphtheria, tetanus and pertussis (DTP), other vaccines have emerged by either adding new components such as *Haemophilus influenzae* vaccine (Hib) or replacing them to improve reactogenicity (50). Similar strategies have been investigated against different respiratory viruses, such as IAV, RSV and SARS-CoV-2. Indeed, several clinical trials have been recently announced, but these vaccines were all applied parentally and no results have been published yet (51–54). Meanwhile, animal studies have elucidated the advantage of using combinatory vaccines against these viral infections. For instance, a single intramuscular injection of a DNA vaccine expressing the IAV-HA and RSV-F proteins induced strong systemic cellular and humoral responses in BALB/c mice, which conferred protection against both pathogens (40). Recent research has also explored the delivery of effective combined IAV/RSV vaccines through the intranasal route (55, 56). The intranasal administration of an adenoviral vector encoding the IAV-HA stem protein in combination with the pre-fusion stabilized form of RSV-F demonstrated in a prime/boost setting the induction of circulating IgG antibodies and protection against both viral infections (55). However, in this approach the local T-cell response is neglected and an important secondary line of defense is missing in case of breakthrough infection. Another prime/boost strategy using a chimeric LAIV-RSV vaccine expressing RSV T-cell epitopes of the RSV-M2–1 protein established antigen-specific CD69^+^CD103^+^ T-cells in the lungs of immunized mice and protect them against IAV and RSV infections (56, 57). Since the licensed LAIV already showed strong age-dependent efficacy most probably due to pre-existing immunity, this might a major obstacle for such a recombinant LAIV approach as well. In contrast, the adenoviral vector platform has been proven to be immunogenic even in the presence of pre-exiting immunity confirmed by successful implantation in the global COVID-19 vaccine campaigns. Furthermore, rare human serotypes or non-human adenoviral vectors could be used to further circumvent this problem (reviewed in (58)). Compared to previous approaches, our mucosal combinatory vaccine was administered in a single-dose schedule and provoked strong systemic and mucosal immune responses including both humoral and cellular effector mechanisms leading to an efficient protection following challenge.

Vaccine immunogenicity and efficacy can be further improved using adjuvants. Indeed, our previous work demonstrated that Ad-IL1β used as a mucosal adjuvant significantly induced vaccine-specific T_RM_ responses and efficacy (33, 34). Despite being a promising candidate as a mucosal adjuvant, the pleiotropic effect of ILlβ may also elicit side effects.

Therefore, we generated two new Ad-expressing TGFβ and CCL17 and investigated their role in combination with the Ad-HA/NP/F vaccine. These cytokines/chemokines were selected due to their function in the development, proliferation as well as differentiation of T-cells (36, 59). TGFβ is an important cytokine for CD8^+^ T_RM_ development, as it is responsible for the induction of CD103 in these cells, which subsequently facilitates their retention in the tissue (23, 35). Furthermore, TGFβ signaling supports the differentiation and function of follicular T helper cells in the draining lymph nodes and the production of IgA and IgG antibodies in the airways of influenza-infected animals (60). In contrast to TGFβ, the beneficial role of CCL17 in promoting mucosal responses was previously observed in a vaccine setting. A DNA plasmid encoding CCL17 was used as an adjuvant in combination with a mucosal DNA vaccine against *Streptococcus mutans* (*S. mutants*). The delivery of CCL17 together with the vaccine antigen improved mucosal IgA and systemic IgG antibody responses and reduced *S. mutans* infection in BALB/c mice (61). Although the mucosal adjuvants, Ad-TGFβ or Ad-CCL17, did not enhance neutralizing antibody responses or the formation of B_RM_, our combinatory mucosal vaccines alone was sufficient to induce B_RM_ as well as mucosal and systemic antibody responses. These findings suggest that this strategy provides a strong humoral immunity to IAV and RSV.

In influenza and RSV human challenge models, mucosal IgA antibodies have an important role in reducing disease severity (62–64). The main source of IgA antibodies in the airway was thought to be plasma cells generated in secondary lymphoid organs, but recent studies have demonstrated that the presence of B_RM_ in the lungs is essential for the rapid generation of IgA-secreting cells upon secondary challenge (27, 65, 66).

In addition to B_RM_, resident T-cell responses are also considered crucial to protect individuals from developing severe respiratory disease. The ability of T-cells to recognize highly conserved internal viral proteins is an advantage against viruses such as influenza, whose surface proteins are antigenically variable (67, 68). Nevertheless, in both influenza- and RSV-infected individuals, T_RM_ were shown to be important for viral clearance and better disease outcome (15, 18, 19, 69). Although none of the mucosal adjuvants showed to enhance humoral responses upon vaccination, antigen-specific CD8^+^ T_RM_ was increased. Furthermore, Ad-TGFβ showed a beneficial effect on the formation of CD4^+^ T_RM_ compared to the non-adjuvanted vaccine. However, the effect of Ad-TGFβ and Ad-CCL17 on the formation of CD8^+^ T_RM_ did not translate into significantly improved vaccine efficacy. Since the non-adjuvanted vaccine is already highly effective in reducing disease severity, a two-fold increase in antigen-specific T_RM_ might not be reflected by reduced viral loads in case of the used high-dose challenges. In this study, we did not formally addressed the contribution of systemic and tissue-resident CD8^+^ T-cells for the heterosubtypic protection against IAV. However, using the same antigen-encoding vectors as a single IAV vaccine, the efficacy was not reduced under FTY720 treatment, which clearly confirmed the essential role of lung-resident lymphocytes (33).

Another relevant factor to take in consideration is the interplay between TGFβ and CCL17 with T_reg_. These cells regulate immune homeostasis and tolerance and this function can impair vaccine-induced immune responses (70). Although we do not have evidence for increased activation of T_reg_ by our adjuvanted combinatory vaccines, the direct link between vaccine-mediated T_reg_ formation and vaccine efficacy is not clear. Nevertheless, a mucosal live-attenuated vaccine candidate against *Streptococcus pneumonia* was shown to induce T_reg_ via the TGFβ pathway with no correlation to low efficacy (71). A more detailed analysis of the immune responses generated after vaccination with Ad-TGFβ and Ad-CCL17 as well as a comparison between the immunogenicity of these mucosal adjuvants and Ad-IL1β would be interesting to better understand the key factors that originated these results.

Independent of the adjuvants, we present here a potent combinatory vaccine for mucosal applications, which provides protection against two of the most relevant respiratory viruses, namely IAV and RSV. This warrants further investigations in regard to translational approaches into the human system.

## Data availability statement

The raw data supporting the conclusions of this article will be made available by the authors, without undue reservation.

## Ethics statement

The study was approved by the Government of Lower Franconia, which nominated an external ethics committee that authorized the experiments. Studies were performed under the project license AZ. 55.2.2-2532-2-1085. The study was conducted in accordance with the local legislation and institutional requirements.

## Author contributions

AVA: Writing – original draft, Writing – review & editing, Conceptualization, Data curation, Formal analysis, Investigation, Methodology, Validation, Visualization. FO: Investigation, Methodology, Writing – review & editing. AS: Investigation, Methodology, Writing – review & editing. VV: Investigation, Methodology, Writing – review & editing. PI: Investigation, Methodology, Writing – review & editing. M-AR-W: Methodology, Resources, Writing – review & editing. WB: Methodology, Resources, Writing – review & editing. DL: Methodology, Supervision, Validation, Writing – review & editing. MT: Conceptualization, Data curation, Funding acquisition, Methodology, Project administration, Supervision, Validation, Visualization, Writing – original draft, Writing – review & editing.
