# Supplementary material for: Filling two needs with one deed: a combinatory mucosal vaccine against influenza A virus and respiratory syncytial virus
Source: Front Immunol. 2024 Jun 21;15:1376395. doi: 10.3389/fimmu.2024.1376395 (PMC11224462; doi:10.3389/fimmu.2024.1376395)
Supplement: Supplementary file 1 [file DataSheet_1.pdf]

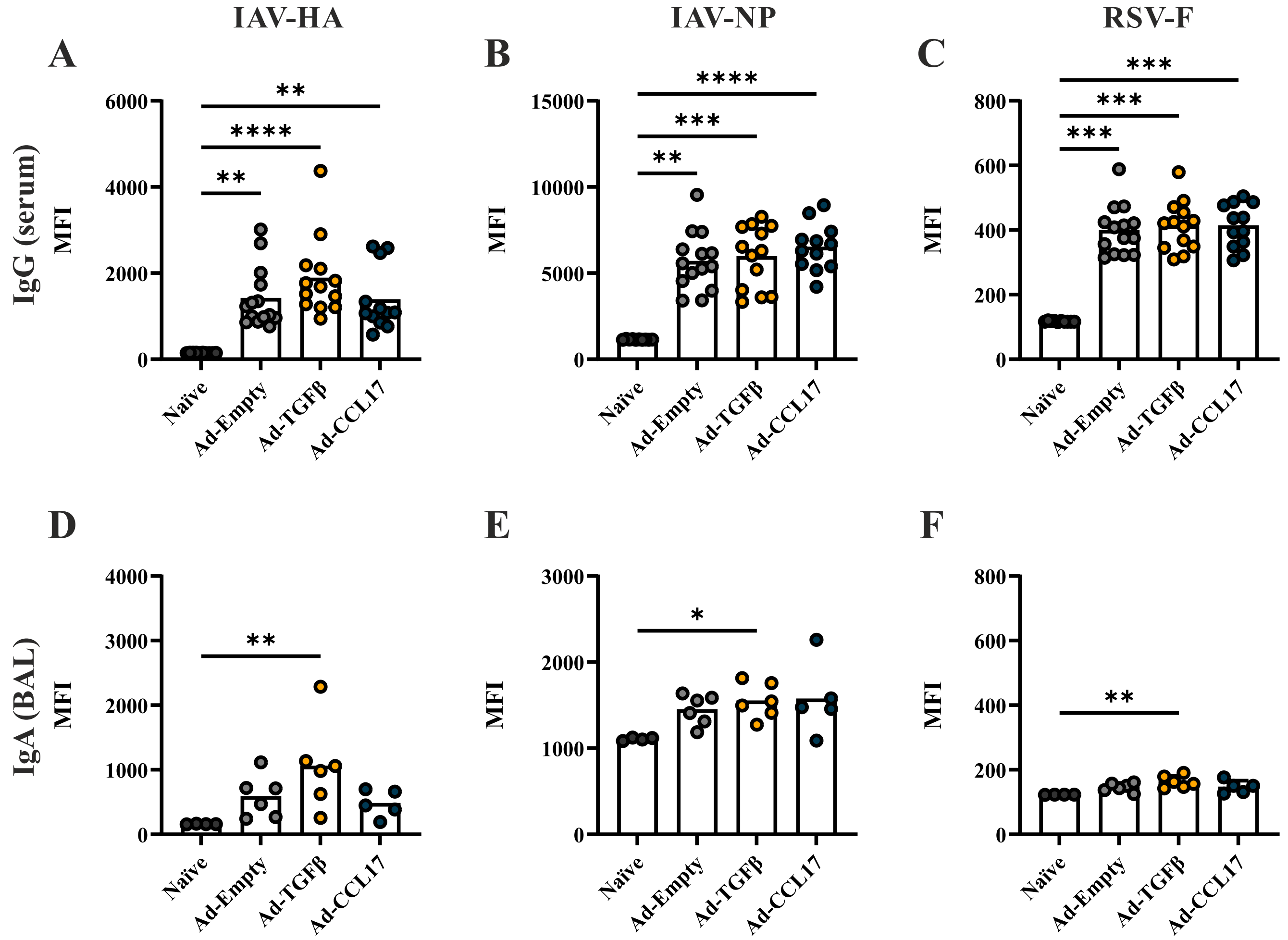

Fig. S1: Systemic and humoral antibody responses after vaccination. BALB/c mice were immunized as described before. The detection of HA-, NP- or F-specific IgG and IgA antibodies in the serum (A,B,C, dilution 1:400) and BAL (D,E,F, dilution 1:10) of immunized animals was performed by a FACS-based antibody assay. HEK 293A cells producing either PR8-derived HA (A,D), NP (B,E) or RSV-derived F protein (C,F) were incubated with the samples, followed by the detection of IgG-specific antibodies with anti-mouse IgG-FITC or IgA-specific antibodies with anti-mouse IgA-FITC. Results represent 10 to 14 mice per group for serum samples and four to six for BAL samples. Each dot represents an individual animal and bars the corresponding mean. Statistical significances were analyzed by Kruskal Wallis Test with Dunn´s multiple comparisons test. *$p<0.05$; **$p<0.01$; ***$p<0.001$; ****$p<0.0001$

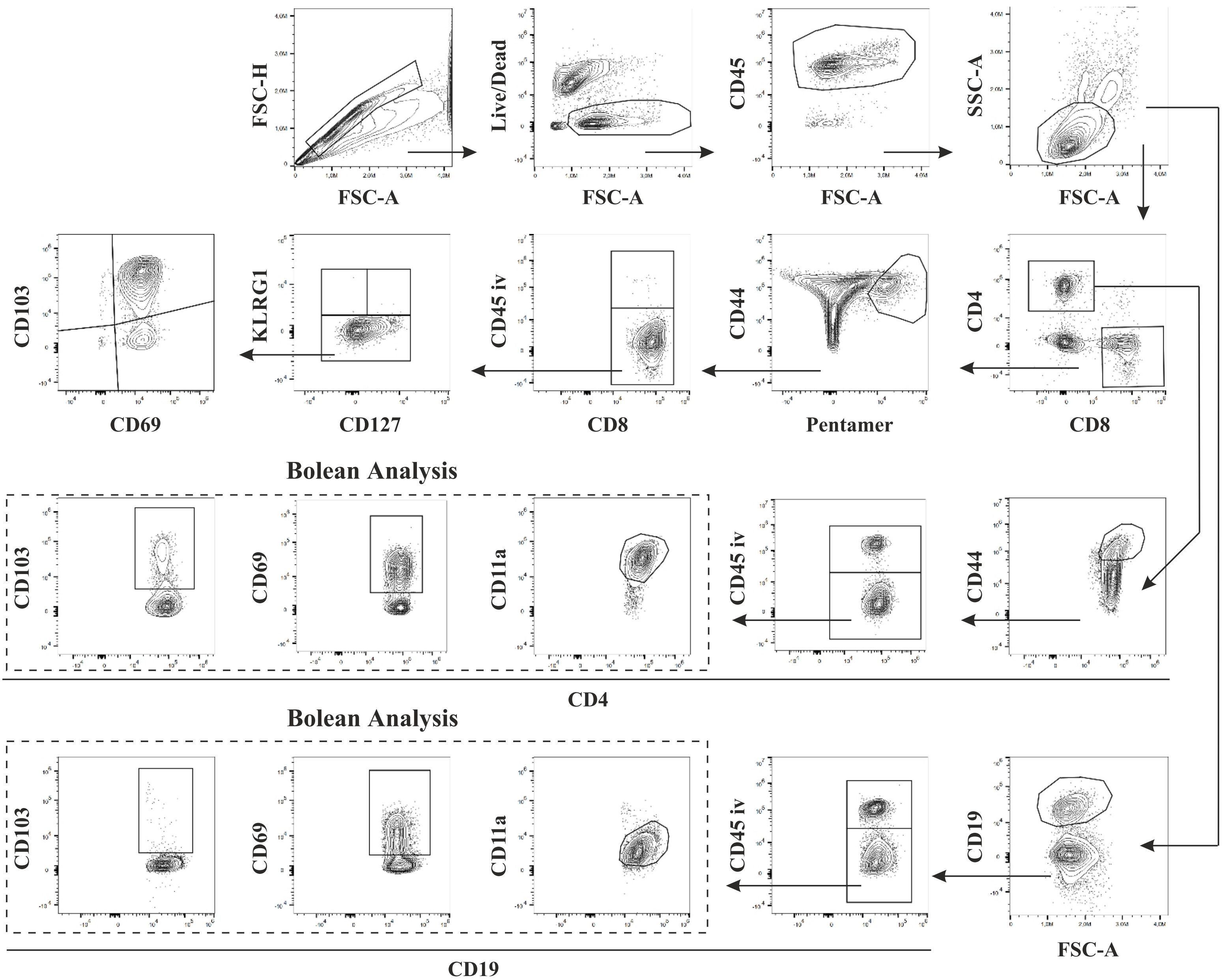

Fig. S2: Gating strategy for phenotypical analysis of memory T- and B-cells in the lungs. BALB/c mice were immunized with 2x10[8] vp of Ad-HA, Ad-NP and Ad-F plus 1x10[9] vp of either Ad-Empty, Ad-TGFβ or Ad-CCL17. Representative gating strategy to characterize the different immune populations in the lungs are indicated by the arrows. Boolean analysis was used to investigate the different resident memory phenotypes in CD4^{+} T- and B-cells.

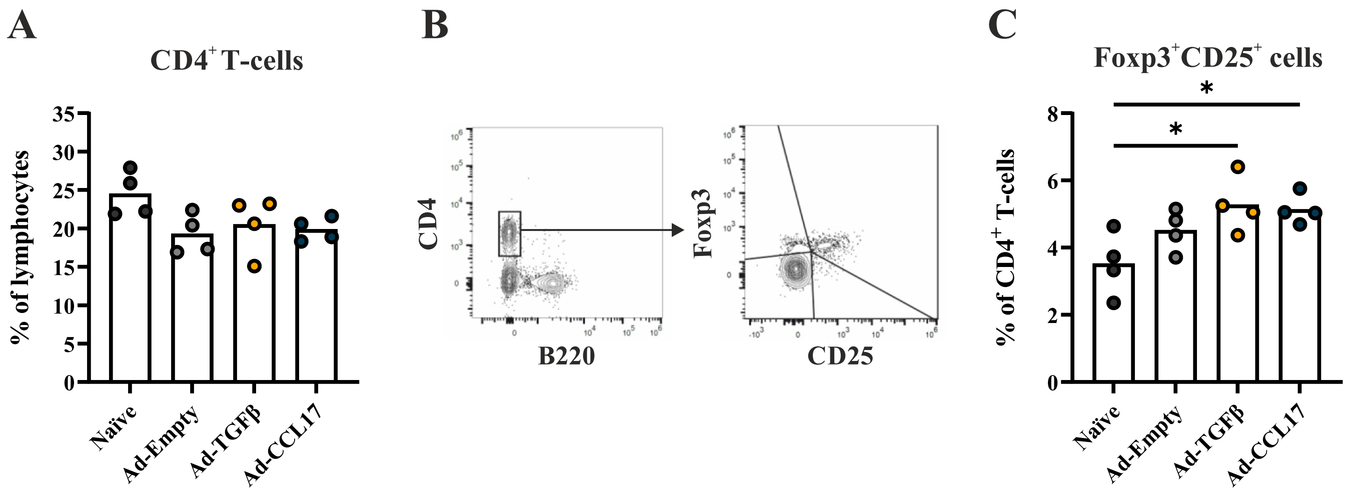

Fig. S3: Phenotypic analysis of lung Foxp3$^+$CD25$^+$CD4$^+$ T-cells. Mice were immunized as described before and 56 days later, lymphocytes were isolated from lung tissue. The percentage of CD4$^+$ T-cells was determined by CD45 and CD4 staining (A). Foxp3 and CD25 were used to characterize the Treg population in CD4$^+$ T-cells (B). The frequency of Foxp3$^+$CD25$^+$ cells was determined in total CD4$^+$ T-cells (C). Each dot represents an individual animal and bars the corresponding mean. Statistical significances were analyzed by one-way ANOVA followed by Tukey's post test. *$p<0.05$

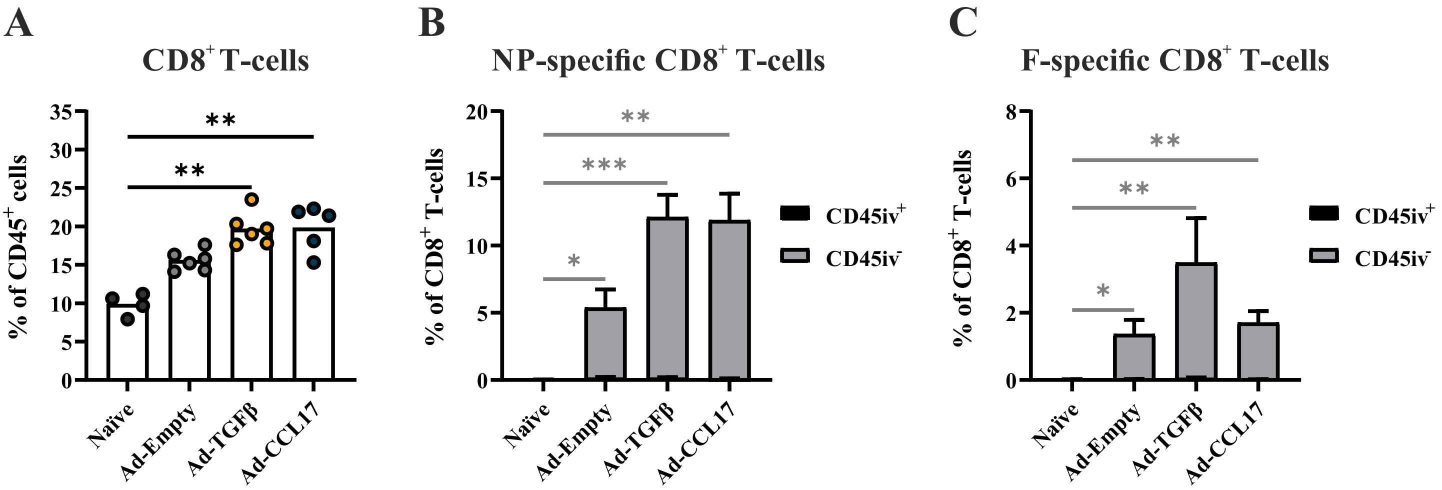

Fig. S4: Phenotypic analysis of lung CD8$^+$ T-cells. Mice were immunized as described before and 56 days later, lymphocytes were isolated from lung tissue. CD8$^+$ T-cells were identified by CD45 and CD8 staining. The graph represents the percentage of CD8$^+$ T-cells within the CD45$^+$ cells (A). Anti-CD45 intravenous staining, described before, was used to distinguish circulating (CD45 iv$^+$) from resident (CD45 iv$^-$) cells in NP- (B) and F- (C) specific CD8$^+$ T-cells. Each dot represents an individual animal and bars the corresponding mean. Statistical significances were analyzed by Kruskal Wallis Test with Dunn´s multiple comparisons test. *$p<0.05$; **$p<0.01$; ***$p<0.001$

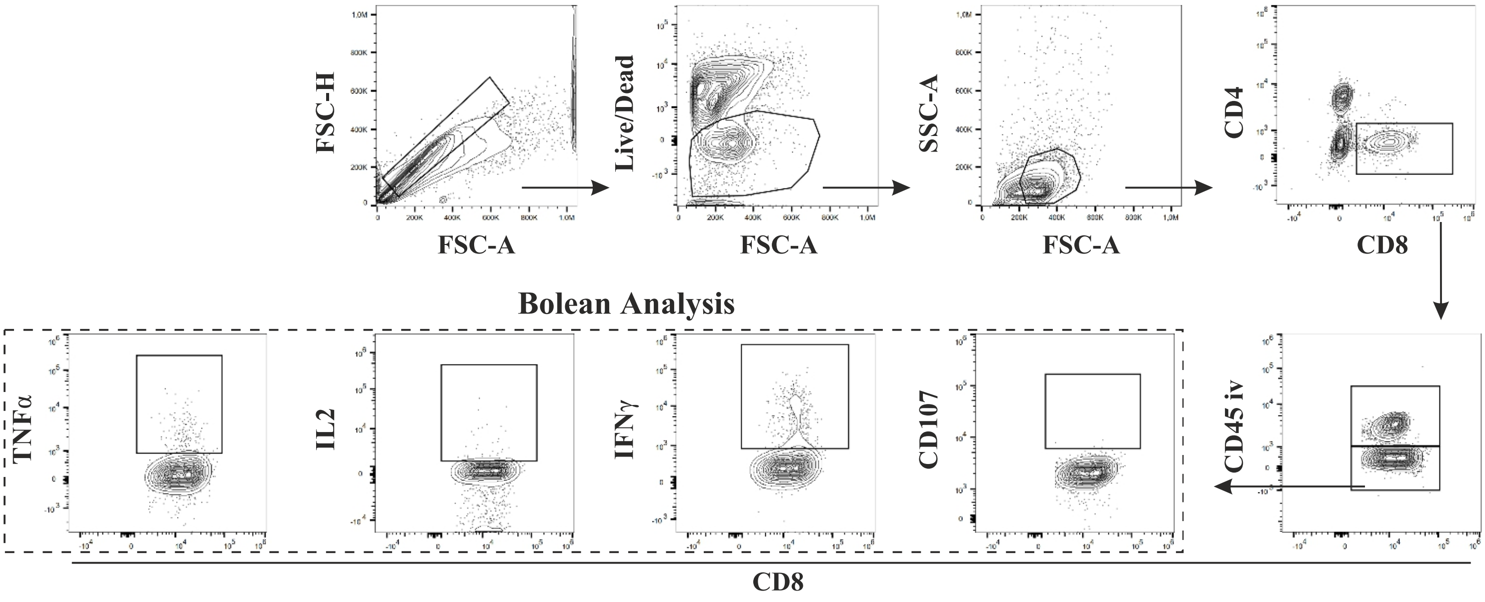

Fig. S5: Gating strategy for functional analysis of CD8$^+$ T-cells in the lungs. BALB/c mice were immunized as described before. Representative gating strategy for the analysis of CD8$^+$ T-cells functionality after *in vitro* restimulation.
